# Immunization with a suicidal DNA vaccine expressing the E glycoprotein protects ducklings against duck Tembusu virus

**DOI:** 10.1186/s12985-018-1053-0

**Published:** 2018-09-14

**Authors:** Jingyu Tang, Zhuangli Bi, Mingyang Ding, Dongdong Yin, Jie Zhu, Li Zhang, Qiuhong Miao, Yingqi Zhu, Guijun Wang, Guangqing Liu

**Affiliations:** 1 College of Animal Science and Technology, Anhui Agricultural University, Hefei, 230036 China; 2 Shanghai Veterinary Research Institute, Chinese Academy of Agricultural Sciences, No. 518 Ziyue Rd, Shanghai, 200241 China; 3 Anhui Province Key Laboratory of Veterinary Pathobiology and Disease Control, Hefei, 230036 China

**Keywords:** Suicidal DNA vaccine, Duck tembusu virus, E glycoprotein, Ducklings

## Abstract

**Backgroud:**

Duck Tembusu virus (DTMUV), a pathogenic flavivirus, emerged in China since 2010 and causing huge economic loss in the Chinese poultry industry. Although several vaccines have been reported to control DTMUV disease, few effective vaccines are available and new outbreaks were continuously reported. Thus, it is urgently to develop a new effective vaccine for prevention of this disease.

**Methods:**

In this study, a suicidal DNA vaccine based on a Semliki Forest virus (SFV) replicon and DTMUV E glycoprotein gene was constructed and the efficacy of this new vaccine was assessed according to humoral and cell-mediated immune responses as well as protection against the DTMUV challenge in ducklings.

**Results:**

Our results showed that the recombinant SFV replicon highly expressed E glycoprotein in DEF cells. After intramuscular injection of this new DNA vaccine in ducklings, robust humoral and cellular immune responses were observed in all immunized ducklings. Moreover, all ducklings were protected against challenge with the virulent DTMUV AH-F10 strain.

**Conclusions:**

In conclusion, we demonstrate that this suicidal DNA vaccine is a promising candidate facilitating the prevention of DTMUV infection.

## Background

Since April 2010, outbreaks of infectious disease in ducks resulting from duck Tembusu virus (DTMUV) have reported throughout the main duck-producing regions of China [1, 2]. Ducks infected with DTMUV often develop high fever, diarrhea, and weight loss, and exhibit retarded growth and decreased egg-laying [1–3]. And the morbidity rate of DTMUV disease can reach up to 90%, as well as the mortality rates vary from 5 to 30% [1, 2, 4, 5]. In addition to China, a similar DTMUV disease has also emerged in Thailand and Malaysia in 2012 [6, 7]. Due to the highly contagious nature of this virus, DTMUV has resulted in huge economic losses in the duck industry. Notably, it has also been reported that DTMUV has a wide host spectrum such as ducks, geese [8], chickens [9] and sparrows [10]. It is noteworthy that Tang et al. reported DTMUV also caused human infection and may be a zoonotic pathogen in China [11]. Thus, a safe and efficient vaccine be developed to prevent this disease is essential.

To date, two types of immunization strategies have been adopted to protect against DTMUV infection. The first involves the vaccination of breeder ducks before they lay eggs, which can result in protection of the offspring via maternally-derived antibodies present in the egg yolk. The second strategy involves the vaccination of susceptible ducklings. While an efficient live attenuated candidate vaccine against DTMUV has been reported [12], this type of vaccine is inefficient for differentiating infected from vaccinated animals (DIVA) and the live attenuated vaccine may hold potential virulence reversion [13, 14]. Moreover, the production of this vaccine is costly and labor-intensive. Therefore, a more cost-effective and safe DTMUV vaccine is urgently needed to protect the duck industry, and to facilitate sero-surveillance and sero-monitoring.

In recent years, suicidal DNA vaccines based on alphavirus replicon vector systems (particularly that of Semliki Forest virus, SFV) have been exploited for gene therapy and vaccine development [15–17]. Indeed, researchers have successfully utilized suicidal DNA vaccines to target a variety of viruses, including those causing influenza in humans [18], peste des petits ruminants virus [19], porcine reproductive and respiratory syndrome virus [20], foot-and-mouth disease [21], and classical swine fever [22]. Suicidal DNA vaccines have many advantages as a strategy for antiviral vaccine development. For example, suicidal DNA vaccines can induce high-level humoral and cell-mediated immunity against various antigens [23–25], and can be used to overcome immunological tolerance by activating innate antiviral pathways [26]. Moreover, efficient immune responses can be obtained with a small amount of plasmid since its self-replicating ability [23], and such suicidal DNA vaccines can also be used for differentiating infected from vaccinated animals (DIVA) [27]. Notably, such vaccines are easy to construct, and have been shown to exhibit efficient expression of interest gene in vitro and to induce humoral and cellular immune responses in vivo, making them an appealing tool for the development of a vaccine against viral diseases [23, 25, 28]. Most importantly, the apoptotic mechanism of these vaccines eliminates the risk of integration of viral DNA into the host cell genome, which is useful to biosafety [29]. All these advantages make suicidal DNA vaccine an outstanding strategy to control DTMUV infection.

DTMUV is a member of genus *Flavivirus*, in family *Flaviviridea*, that harbors a single-stranded positive-sense RNA genome comprised of a single large open reading frame (ORF), approximately 10,990-nucleotides in size, which encodes three structural proteins including capsid protein (C), pre-membrane (prM) and envelope (E) glycoprotein and seven nonstructural (NS) proteins including NS1, NS2A, NS2B, NS3, NS4A, NS4B, and NS5 [30, 31]. E glycoprotein is embedded at the viral surface and was shown to be the major target of neutralizing antibodies that clear *Flavivirus* infections [32]. Moreover, previous studies of flaviviruses such as West Nile virus [33], Japanese encephalitis virus [34], and tick-borne encephalitis virus [35] indicated that the gene encoding this E protein might be useful for vaccine development. For example, Wang et al. reported that mice immunized with the recombinant E protein which was expressed and purified form *Escherichia coli* developed E protein antibodies and were protected from infection with West Nile virus [36]. A previous study showed that immunization with pseudotype baculovirus expressing E protein of Japanese encephalitis virus in mice could elicit high levels of protective immunity [37]. Similarly, Raviprakash et al. found the candidate DNA vaccine expressing E protein of dengue virus type 1 produced dengue-1 specific antibodies that were both neutralizing and long lasting in mice [38]. All these previous studies suggest that the E glycoprotein could also be a good vaccine candidate against the DTMUV infection.

In this study, we constructed a suicidal DNA vaccine expressing the E glycoprotein of DTMUV. The efficacy of this potential vaccine was further evaluated according to humoral and cell-mediated immune responses as well as protection against the DTMUV challenge in ducklings.

## Methods

### Cell culture and virus propagation

Duck embryo fibroblasts (DEF) were prepared from 12-day-old SPF duck embryos (Harbin Veterinary Research Institute, Harbin, China) according to standard protocols. Cells were seeded at a density of 10^6^ cells/ml in Dulbecco’s Modified Eagle Medium (DMEM; GIBCO), supplemented with 10% fetal bovine serum (FBS; GIBCO), 100 units/ml penicillin (GIBCO), and 100 mg/ml streptomycin (GIBCO), in an incubator at 37 °C and 5% CO_2_. DTMUV virus AH-F10 is a virulent strain isolated during an outbreak in Anhui province, China in 2013.

### Suicidal DNA vaccine construction

Total DTMUV RNA was extracted from a viral suspension using a TIANamp Viral Total Nucleic Acid Purification Kit (TIANGEN Biotech, Beijing, China), according to the manufacturer’s instructions. First-strand complementary DNA (cDNA) was then synthesized using M-MLV reverse transcriptase (Promega, Madison, WI, USA) and 6-nt random primers (Promega), and the ORF of the E gene was amplified by reverse transcription (RT)-PCR using the following primers: 5′- CGGGATCCGCC ACCATGTTCAGCTGTCTGGGGATG C-3′ and 5’-TCCCCCGGGGGCATTGACATTTACTGCCAG-3′. These primers were designed with *Bam*HI and *Sma*I restriction enzyme sites (underlined) for cloning purposes, as well as a Kozak sequence (italics) to optimize target gene expression. PCR products were purified and subcloned into pSCA1, an SFV replicon-based vector. The composition of the resulting plasmid, referred to as pSCA1-E, was confirmed by PCR and sequencing analyses. The vector was maintained by transforming into competent *Escherichia coli* DH5α cells, and purified using the Wizard Plus Maxiprep DNA Purification System (Promega).

### Detection of E expression in DEF cells

DEF cells were transfected with pSCA1-E using Lipofectamine 2000 reagent (Invitrogen), according to the manufacturer’s instructions. Forty-eight hours post-transfection, the cells were subjected to immunofluorescence microscopy (IFA) and western blot analyses for evaluation of the expression of E protein. For IFA analysis, cell monolayers were cultured on cover slips and fixed by incubation with cold 100% acetone for 30 min at − 20 °C. Slides were then incubated with rabbit anti-E polyclonal serum (1:1,000) at 37 °C for 30 min, followed by fluorescein-conjugated goat anti-rabbit antibodies (Beijing Zsbio, Beijing, China) at 37 °C for 30 min. Cells were visualized using a Leica fluorescence microscope (Olympus, Tokyo, Japan).

For western blot analyses, DEF cells were transfected with pSCA1-E, cultivated for 48 h, and lysed by incubation with lysis buffer. Proteins were then separated by 10% sodium dodecyl sulphate polyacrylamide gel electrophoresis (SDS-PAGE) and transferred to nitrocellulose membranes (Bio-Rad, Hercules, CA, USA). Membranes were incubated with rabbit anti-DTMUV E protein polyclonal serum (1:1,000), followed by HRP-conjugated goat anti-rabbit IgG antibodies (Beijing Zsbio). Protein bands were visualized by 3, 3′-diaminobenzidine tetrahydrochloride staining (TIANGEN).

### Immunization of ducks and virus challenge

Forty female specific-pathogen-free (SPF) Sheldrake ducklings (7-day-old, free of anti-DTMUV antibodies) were obtained from Harbin Veterinary Research Institute, China. The ducklings were randomly divided into four groups of 10 ducklings each, and housed in separate rooms. The animals were then immunized by intramuscular injection of 200 μg pSCA1-E plasmid (pSCA1-E group), 200 μg empty pSCA1 vector (pSCA1 group), 200 μl commercially inactivated DTMUV vaccine (inactivated HB strain group, Rinpu, Tianjin, China) or 200 μl PBS (negative control) into the quadriceps muscle. Immunization was carried out twice at 2-week intervals. Serum samples from the four groups were collected 0, 1, 2, 3, and 4 weeks post-primary immunization (ppi) for specific antibodies and neutralizing antibodies test. At 2 and 4 weeks after primary immunization, bloods were collected for lymphocyte proliferation assay and cytokines detection. Two weeks after the second immunization, ducklings were challenged with 10^4.6^ ELD_50_ (embryo lethal dose) of DTMUV AH-F10 via intramuscular injection. Ducklings were observed daily for 2 weeks post-challenge.

### Antibody responses against DTMUV

Serum samples from ducks were determined by an indirect ELISA test using the recombinant E protein of DTMUV, produced in *E. coli* BL21 (DE3), as antigen. The E protein was expressed in *E. coli* BL21 (DE3) using the PET 32a expression system (Novagen) and the product were purified by dialysis method. Ninety-six wells flat-bottomed plates (Corning Costar) were coated with recombinant E protein in 0.1 M carbonate/bicarbonate buffer (pH 9.6) and incubated overnight at 4 °C. After blocking with 5% BSA in PBS, plates were incubated with duplicate twofold serial dilutions of test sera for 1 h at 37 °C. Rabbit anti-ducks IgG HRP (KPL) at a 1:2000 dilution was then added for 1 h at 37 °C, followed by the addition of the substrate 2 mm Sulfuric acid. Absorbance was determined at 450 nm using a Bio-Rad microtitre plate reader.

### Viral neutralization testing

For detection of virus-neutralizing antibodies (VNAs) against DTMUV, primary duck embryonic fibroblasts (DEFs) prepared from 10-day-old duck eggs (DTMUV-free) were trypsinized, seeded in 75-cm2 flasks, and cultivated in DMEM containing 10% FBS, 2 mM glutamine, 100 U/ml penicillin, and 100 μg/ml streptomycin at 37 °C with 5% CO2. Serum samples were then heat-inactivated at 56 °C for 30 min, and serially diluted (with an initial 1:2 dilution) with culture medium in 96-well plates. Diluted serum samples were pre-incubated with 200 TCID50 (tissue culture infectious dose; infectious dose capable of killing 50% of cultivated cells) DTMUV at 37 °C for 1 h. Mixtures were then added to 96-well plates containing approximately 105 DEFs/well, and antibody titres were determined by monitoring CPE (cytopathic effect) over 6 days. Each sample was independently tested in triplicate.

### Lymphocyte proliferation assay

To evaluate cellular immune responses induced by pSCA1-E, lymphocyte proliferation was evaluated in ducklings at 2 and 4 weeks post immunization. For these analyses, blood samples were collected from the jugular vein in blood collection tubes containing 3.8% sodium citrate. Peripheral blood mononuclear cells (PBMCs) were then isolated by treatment with Ficoll-Paque Plus (Amersham Biosciences, Buckinghamshire, UK) for 20 min at 18 °C, seeded (100 μl volumes; approximately 10^5^ cells/well) in 96-well plates, and mixed with 100 μl of medium supplemented with or without inactivated DTMUV. Concanavalin A (5 μg/ml; Sigma, St. Louis, MO, USA) was used as a positive control. PBMC samples were prepared in triplicate. Proliferation was measured according to a standard protocol. Briefly, after incubation for 96 h, 20 μl of 3-(4, 5-dimethylthylthiazol-2-yl)-5-(3-carboxymethoxyphenyl)-2 -(4-sulfophenyl)-2H tetrazolium (inner salt; Promega) was added to each well, and the cells were incubated for 4 h, after which the optical density of each well was measured at 450 nm (OD_450_). The stimulation index (SI) was calculated as the ratio of the average OD_450_ of wells containing antigen-stimulated cells to that of wells containing cells treated with medium only.

### Cytokine assays

To evaluate the serum levels of the helper T cell (Th) 1- and Th2-type cytokines interferon (IFN)-γ and interleukin (IL)-4, respectively, supernatants were collected from peripheral blood at week 1 and 2 after the first vaccination and week 1 and 2 after the second vaccination, and subjected to enzyme-linked immunosorbent assay (ELISA) analysis using Bio-Plex Pro kits (Bio-Rad), according to the manufacturer’s instructions.

### Detection of viral RNA

Total RNA was extracted from the samples (serum and liver) of ducks with RNAeasy (Qiagen, Germany), and used immediately for cDNA synthesis. cDNA synthesis was performed with SuperScript II reverse transcriptase (RT) (Invitrogen, USA). Then the viral load in the tissues of ducklings was detected using qRT-PCR assay. The primers were designed referring to the sequences of E gene of DTMUV AH-F10. The forward and reverse primers were 5′- ATGTTCAGCTGTCTGGGGATGC-3′ and 5’-GGCATTGACATTTACTGCCAG-3′, respectively.

### Histopathological analysis of organs

At 2 weeks post-challenge, all ducklings were bled and euthanized. Organs were collected and subjected to histopathological analysis. After dehydration, organ tissue blocks were embedded in paraffin and cut into 4-μm thick sections. Sections were then stained with hematoxylin and eosin and observed under a microscope (Olympus Corporation, Tokyo, Japan).

### Statistical analysis

Statistical analysis was performed by using SPSS 15.0 (SPSS, Chicago, IL, USA). All data were presented as the mean ± standard deviation (SD). The comparison between data was calculated using Student’s t-test (between two groups) and one-way ANOVA and Tukey’s multiple comparison tests (between multiple groups). Differences were considered statistically significant when *P* values < 0.05. Survival curves were made using the Kaplan-Meier method, and analyzed with a Long-rank (Mantel-Cox) test.

## Results

### Expression of E glycoprotein in DEF cells

**Fig. 1:**
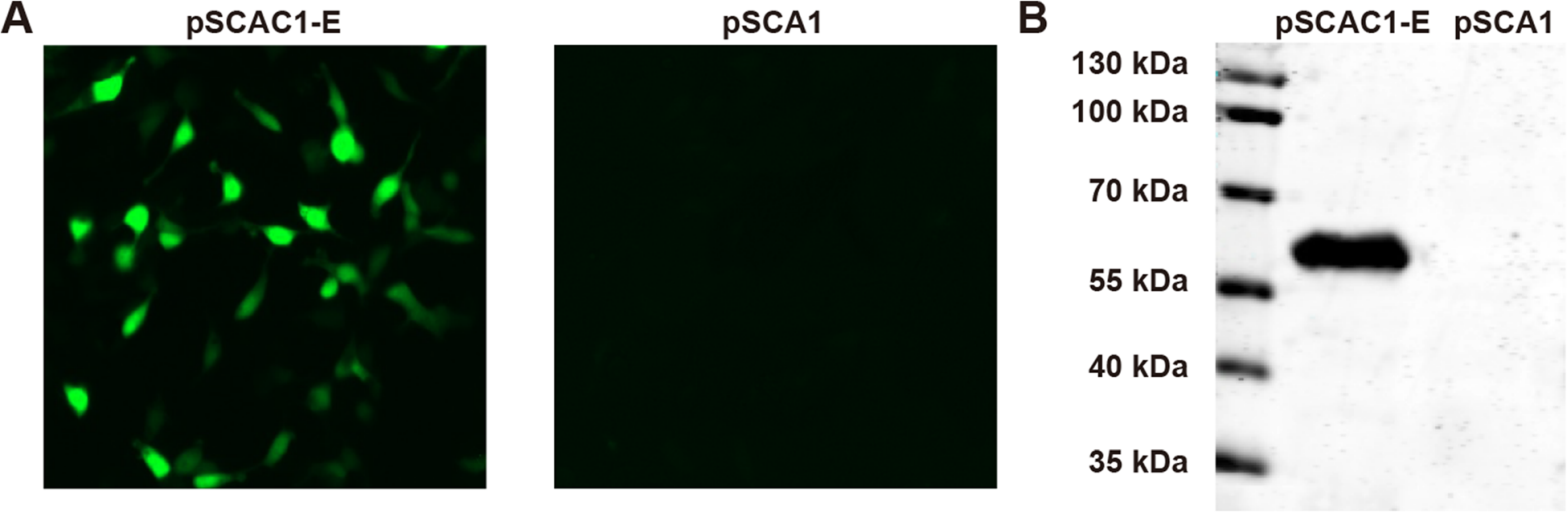
Evaluation of in vitro envelope (E) glycoprotein expression by immunofluorescence assay (IFA) and western blot analysis. **a** DEF cells were transfected with pSCA1-E or the negative control plasmid pSCA1. At 48 h post-transfection, cells were stained using rabbit anti-E polyclonal antiserum and a FITC-conjugated goat anti-rabbit IgG, and observed with a fluorescence microscope. **b** Western blot analysis of E glycoprotein expression. Lane 1, Pre-stained protein marker; lane 2, lysate generated from DEF cells transfected with pSCA1-E plasmid; lane 3, lysate generated from DEF cells transfected with empty pSCA1 vector

To test whether the constructed plasmid (pSCA1-E) could express E glycoprotein in vitro, DEF cells were transfected with individual plasmids and the expression of E glycoprotein was examined via IFA and western blot analyses. As expected, cells transfected with pSCA1-E exhibited a robust green fluorescence signal. In contrast, no fluorescence signal was observed in control cells transfected with pSCA1 empty plasmid (Fig. 1a). Meanwhile, western blot analysis detected a protein approximately 55 kDa in size in the lysates of cells transfected with pSCA1-E, but not in those of the control cells (Fig. 1b). These data demonstrate that E glycoprotein can be highly expressed by pSCA1-E plasmid and retained its high immunoreactivity of the anti-E antibody in vitro.

### Humoral immune responses in ducklings immunized with pSCA1-E plasmid

**Fig. 2:**
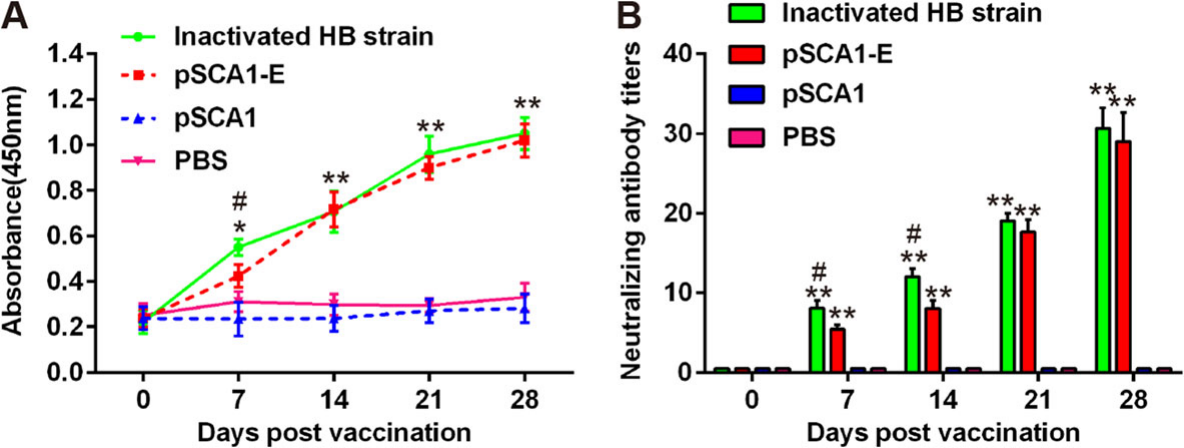
Antibody levels in ducklings after immunization. **a** E glycoprotein-specific antibody levels were detected by indirect ELISA analysis. **b** Neutralization antibody levels were detected by serum neutralization assay. Each data represents the mean ± SD. *P < 0.05, **P < 0.01 vs. PBS group; #*P* < 0.05 vs. inactivated HB strain group

To evaluate the immunogenicity of the recombinant replicon plasmid pSCA1-E, it was inoculated into ducklings as described in Methods. Blood was collected at week 0, 1, 2, 3 and 4 after the first vaccination to test for the presence of E glycoprotein-specific antibodies and neutralization antibodies. Specific anti-E antibody level was determined by an indirect ELISA. As shown in Fig. 2a, after the primary immunization, E glycoprotein-specific antibodies were detected in all ducklings immunized with pSCA1-E plasmid. After the booster immunization, the antibody level increased rapidly, and significantly higher than control groups (pSCA1 and PBS group). Meanwhile, the inactivated HB strain vaccine group also exhibited a high antibody level, whereas there was no significant difference between the pSCA1-E group and inactivated HB strain group. Next, a microneutralization assay was performed to evaluate the ability of serum samples to neutralized DTMUV virulent strain. As shown in Fig. 2b, all ducklings immunized with pSCA1-E plasmid and inactivated HB strain vaccine developed neutralizing antibodies after first immunization and reached peak level at 4 weeks. Conversely, detectable levels of neutralizing antibodies were not produced by any of the ducklings in the pSCA1 control and PBS negative control groups during the experiment.

### DTMUV-specific T cell proliferation

**Fig. 3:**
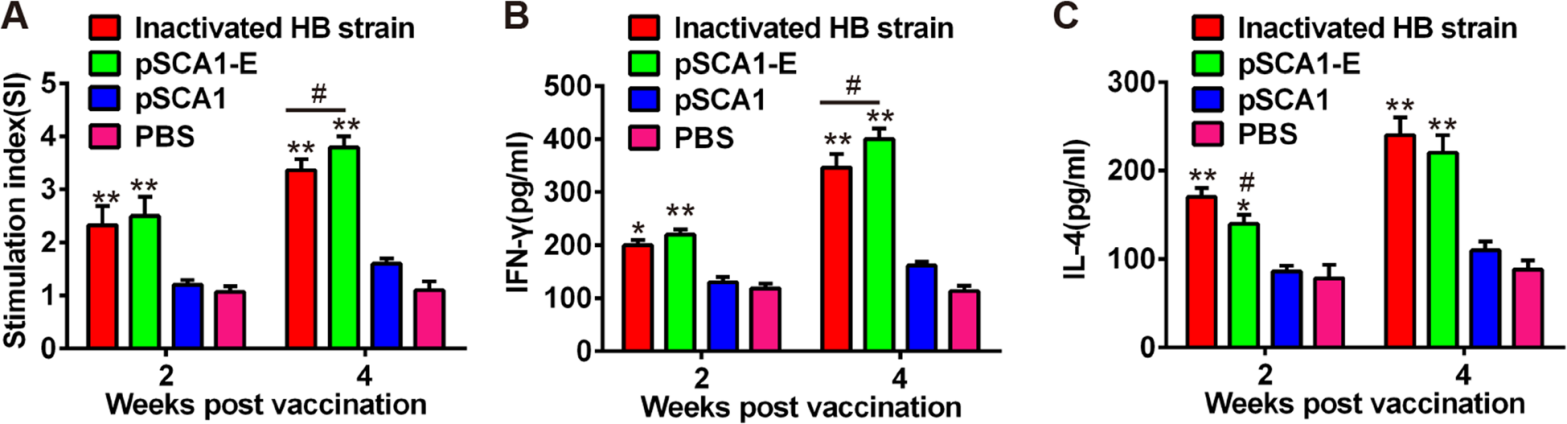
Cellular immune response in ducklings immunized with pSCA1-E plasmid. **a** Specific proliferation of PBMCs in immunized ducks. The data are presented as the mean absorbance value of each group. **b** The interferon gamma (IFN- γ) concentration in serum from immunized ducklings was measure by ELISA. **c** The interleukin (IL-4) concentration in serum from immunized ducklings was measure by ELISA. Each data represents the mean ± SD. *P < 0.05, **P < 0.01 vs. PBS group; #*P* < 0.05 vs. inactivated HB strain group

To investigate cellular immunity responses induced by pSCA1-E plasmid, we analyzed the lymphocyte proliferative responses of all ducks at 2 and 4 weeks after the primary immunization. The results manifested that pSCA1-E and inactivated HB strain vaccine displayed obvious and intense lymphocyte proliferate responses (Fig. 3a). Notably, at 4 weeks, the values of pSCA1-E immunization group were higher than inactivated HB strain vaccine group, suggesting the robust cellular immune responses in pSCA1-E group.

### Cytokines induced by pSCA1-E immunization

To further evaluate cellular immunity responses induced by pSCA1-E plasmid, serum IFN-γ and IL-4 levels were assessed at 2 and 4 weeks after the primary immunization by ELISA analysis. As shown in Fig. 3b and c, the mean levels of both cytokines were significantly higher in ducklings inoculated with pSCA1-E plasmid than in those treated with pSCA1 or PBS. These were no significant difference between pSCA1-E group and inactivated vaccine group.

### Protection against DTMUV challenge

**Fig. 4:**
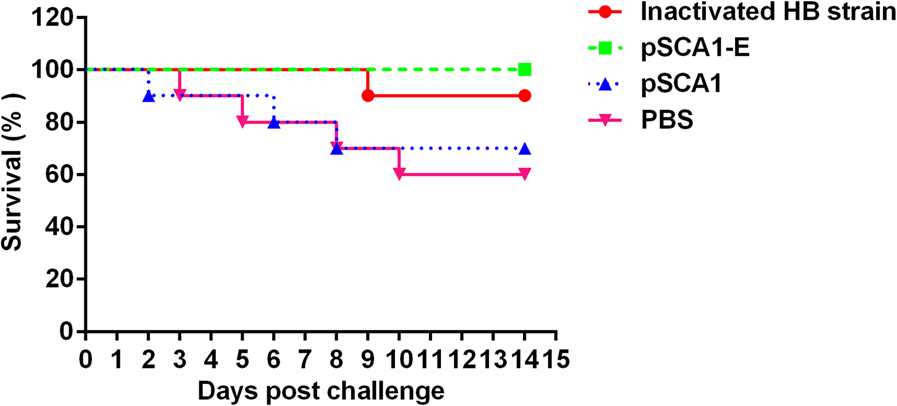
The survival rate in ducklings after virulent DTMUV challenge. The statistical significance of differences in mortality between groups was determined using Kaplan-Meier method, and analyzed with a Log-rank (Mantel-Cox) test. For pSCA1-E group vs. PBS group, *P* < 0.05; for inactivated HB strain group vs. PBS group, *P* < 0.05; and for pSCA1-E group vs. inactivated HB strain group, *P* > 0.05

To determine whether the suicidal DNA vaccine could protect against infection by a virulent DTMUV strain, ducklings were challenged with DTMUV AH-FH10 at 2 weeks after the second immunization. All ducklings were housed in an isolation facility and examined for 2 weeks after the challenge. The ducklings from the pSCA1 and PBS control groups exhibited symptoms typical of DTMUV infections, including the manifestation of neurological signs, weight loss, and green-colored feces, beginning at 2 days post-viral challenge. As showed in Fig. 4, all of the ducklings were protected against challenge with virulent DTMUV in pSCA1-E group and 9 of 10 ducklings were protected in inactivated HB strain vaccine group. However, 3 and 4 of the 10 ducklings in pSCA1group and PBS group died respectively.

### Detection results of viral RNA in blood and organs

**Fig. 5:**
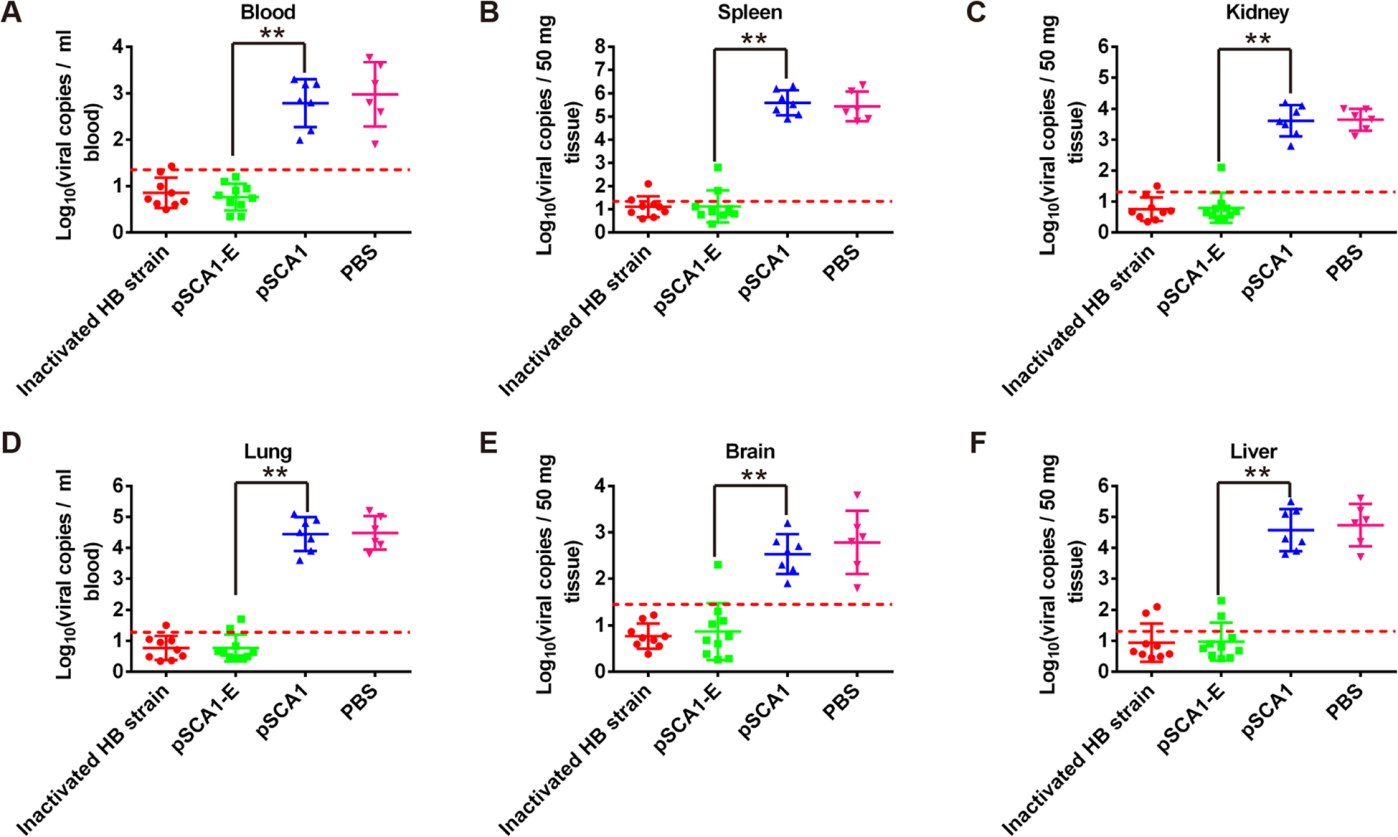
Quantification of viral loads in blood and organs of euthanized ducklings using real-time qRT-PCR. **a** Viral loads in blood. **b-f** Viral loads in spleen, kidney, lung, brain and liver. The dotted line marks the positive cut-off. **P < 0.01 vs. pSCA1 group

In order to detect viral load in blood and organs of ducklings after the virus challenge, real-time qRT-PCR was performed using E gene-specific primers. As expected, high viral copies were detected in blood (Fig. 5a), spleen (Fig. 5b), kidney (Fig. 5c), lung (Fig. 5d), brain (Fig. 5e) and liver (Fig. 5f) of all ducklings in pSCA1 and PBS group. However, only one or two ducklings were positive in pSCA1-E group and inactivated HB strain vaccine group. Importantly, the viral loads in these positive samples were also significantly lower than two control groups (Fig. 5a-f). These data indicated that vaccination with pSCA1-E could significantly reduce the onset of viremia and decrease virus replication in ducklings.

### Histopathological assays of major target organs in DTMUV-infected ducklings

**Fig. 6:**
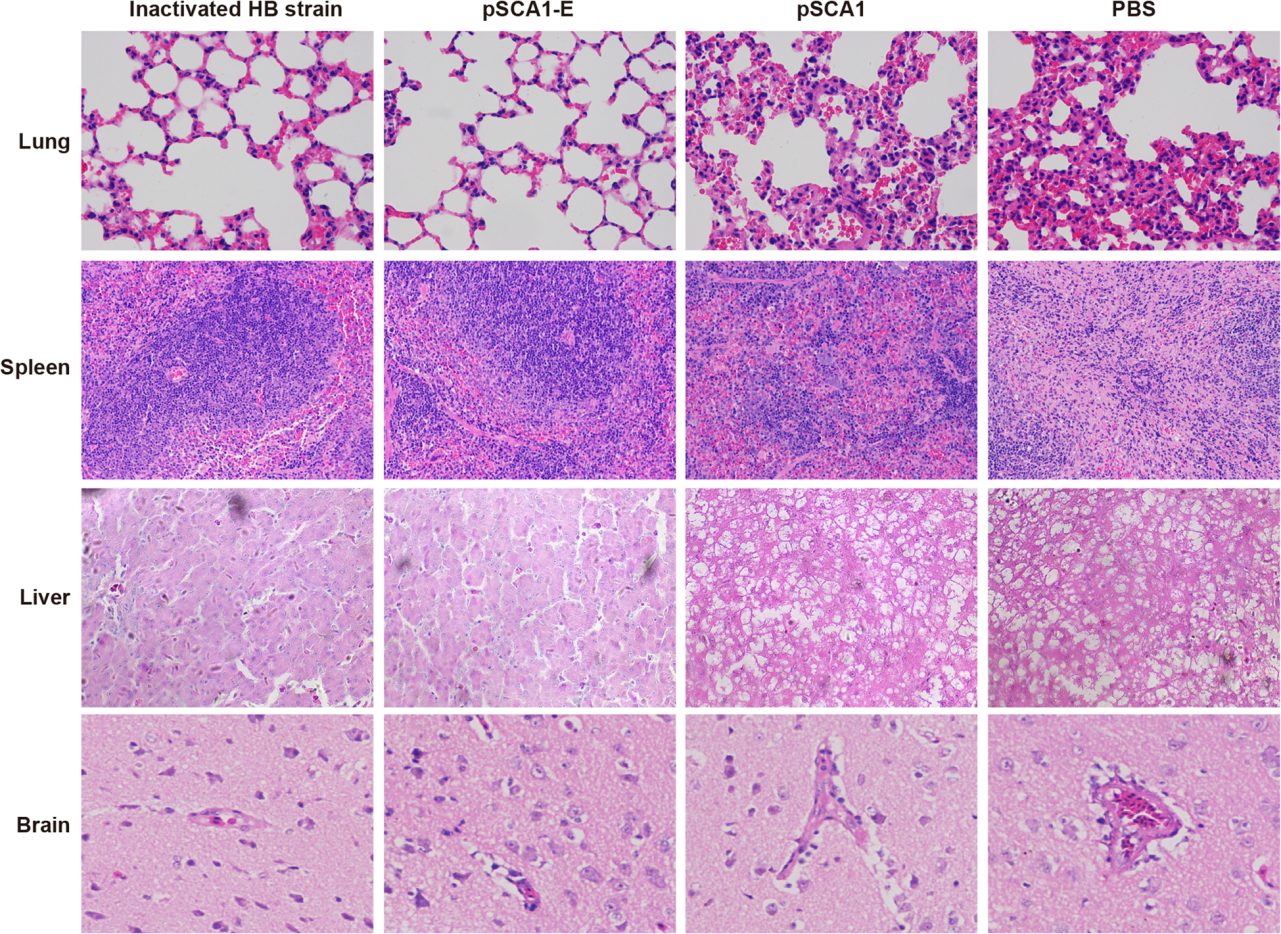
Histopathologic changes of ducklings after DTMUV infection. Groups of 10 ducks were intramuscularly challenged with 10^4.6^ ELD_50_ of the DTMUV AH-F10 strain at 2 weeks after the second immunization. At 2 weeks post-challenge, all ducklings were bled and euthanized and their ovaries (lung, spleen, liver and brain) were collected for histopathologic examination

To further understand the basis for the pSCA1-E vaccine protection of ducklings, we examined the histopathological changes of major target organs. As shown in Fig. 6, histopathological changes were observed in the organs of the ducklings in the non-immune groups (pSCA1 and PBS group) including interstitial pneumonia and pneummorrhagia in the lung, the number of lymphocytes in the white pulp decreased significantly and the boundaries between the red pulp and white pulp disappeared in the spleen, hepatocyte vacuolation and heterophilic granulocyte infiltrate in the liver and encephalitic lesions with severe perivascular inflammatory infiltrates in the brain. However, the organs of the ducklings in the two vaccinated groups showed no histologic lesions.

## Discussion

The E glycoprotein of *Flavivirus* is the major antigen targeted by neutralizing antibodies, and several studies have shown that E protein-derived vaccines can protect animals against West Nile and Japanese encephalitis viruses [39, 40]. In this study, we therefore generated a suicidal DNA vaccine expressing the E gene of DTMUV. IFA and western blot analyses demonstrated that the E protein was highly expressed in DEF cells, while immunization analyses suggested that the DNA vaccine induced strong humoral and cellular immune responses in ducklings. More importantly, this DNA vaccine conferred 100% protection against the virulent DTMUV challenge in ducklings. In any case, histopathological changes were observed in the organs of the ducklings in the non-immune groups including interstitial pneumonia and pneummorrhagia in the lung, the number of lymphocytes in the white pulp decreased significantly in the spleen, hepatocyte vacuolation in the liver and encephalitic lesions in the brain. Conversely, the organs of the ducklings inoculated with pSCA1-E appeared healthy and normal (data not shown). These findings indicate that the pSCA1-E vaccine provides well protection against DTMUV infection in ducklings.

It has become increasingly accepted that the IFN-γ dependent Th1 cellular response is required for the protection against Flavivirus infection [41–43]. In this study, we also found the ducklings immunized with pSCA1-E exhibited distinct robust Th1 cellular immune responses than the negative control animals. This effect could be due to the production of double-stranded viral RNA-derived intermediates induced by the suicidal DNA vaccine, which have been shown to induce caspase-dependent apoptosis in transfected cells [44]. These apoptotic cells could then have been taken up by dendritic cells [45], which can act as an adjuvant for T cell-specific stimulation by the antigen [44]. The intensity of the host response to suicidal DNA vaccine typically corresponds to the serum levels of pro-inflammatory cytokines such as IFN-γ, which inhibits the viral replication. Meanwhile, ILs are known to mediate T and B lymphocyte activation, proliferation, and differentiation during immune activation and regulation [46, 47]. Our data demonstrated that the pSCA1-E vaccine also elicited a higher level of IL-4 in ducklings. These findings confirmed that pSCA1-E vaccine induces an enhanced Th1-type response and cell-mediated immune responses may play an important role in this DNA vaccine against DTMUV infection.

## Conclusion

In conclusion, the pSCA1-E suicidal DNA vaccine induced strong humoral and cellular immune responses as well as completely resisted to DTMUV challenge in ducklings. These findings suggest that our suicidal DNA vaccine might comprise an effective prophylactic measure against DTMUV infection in ducklings. Further work is required to evaluate the protective effects of this DNA vaccine in field tests.

## Abbreviations

C: Capsid protein
cDNA: Complementary DNA
CPE: Cytopathic effect
DIVA: Differentiating infected from vaccinated animals
DMEM: Dulbecco’s Modified Eagle Medium
DTMUV: Duck Tembusu virus
E: Envelope glycoprotein
ELD_50_: 50% embryo lethal dose
FBS: Fetal bovine serum
IFN-γ: Interferon (IFN)-γ
NS: Nonstructural proteins
ORF: Open reading frame
prM: Pre-membrane
SDS-PAGE: Sulphate polyacrylamide gel electrophoresis
SFV: Semliki Forest virus
SPF: Specific-pathogen-free
VNAs: Virus-neutralizing antibodies
